# Autologous pericardium used for reconstruction of left innominate vein in patient with mediastinal venous aneurysm:a case report

**DOI:** 10.1186/s12893-020-00749-0

**Published:** 2020-05-05

**Authors:** Cheng Fang, Hui Pan, Zhoubin Li, Liang Ma, Weili Han

**Affiliations:** 1 Department of Lung transplantation, The First Affiliated Hospital, Zhejiang University School of Medicine, No.79 Qingchun Road, Hangzhou, 310003 China; 2 Department of Cardiothoracic surgery, The First Affiliated Hospital, Zhejiang University School of Medicine, No.79 Qingchun Road, Hangzhou, 310003 China

**Keywords:** Mediastinal mass, Venous aneurysm, Pericardium, Left innominate vein, Case report

## Abstract

**Background:**

Mediastinal venous aneurysm is a very rare disease and can be easily misdiagnosed. Patients are often asymptomatic while venous aneurysm of large size with adjacent structures oppressed can lead to discomfort. The surgical treatment for aneurysm of large vessels is often complex and challenging.

**Case presentation:**

We reported a 52-year-old man with mediastinal mass who received operation on July 2019 in our hospital. Left innominate vein aneurysm was diagnosed during operation with superior vena cava involved. The aneurysm was resected and pericardium was taken to repair part wall of superior vena cava and reconstruct left innominate vein. The patient’s postoperative course was uneventful.

**Conclusions:**

Venous aneurysm should be considered when mediastinal mass has no clear boundary with large veins or even seems to connect with them. Magnetic resonance imaging, computed tomographic angiography and invasive venography can be performed to further evaluate the mass once diagnosis of venous aneurysm was suspected. Using pericardium to repair large veins is a good choice which is safe and costless.

## Background

Mediastinal venous aneurysm is a very rare disease [1] and often difficult to be distinguished from other mediastinal tumors, which may lead to misdiagnosis [2]. They are often asymptomatic. However, venous aneurysm of large size with adjacent structures oppressed can cause clinical symptoms such as cough, chest pain, hemoptysis and dyspnea [3]. The etiology of mediastinal venous aneurysms remains undetermined, including congenital malformations, trauma, inflammation, infection and degenerative change in the vessel wall [4]. There are several literatures on the surgical treatment of venous aneurysm, however, none of them use pericardium. Herein, we report a case of left innominate venous aneurysm who received operation in our hospital. Pericardium was used to repair part wall of the superior vena cava (SVC) and reconstruct the left innominate vein.

## Case presentation

**Fig. 1:**
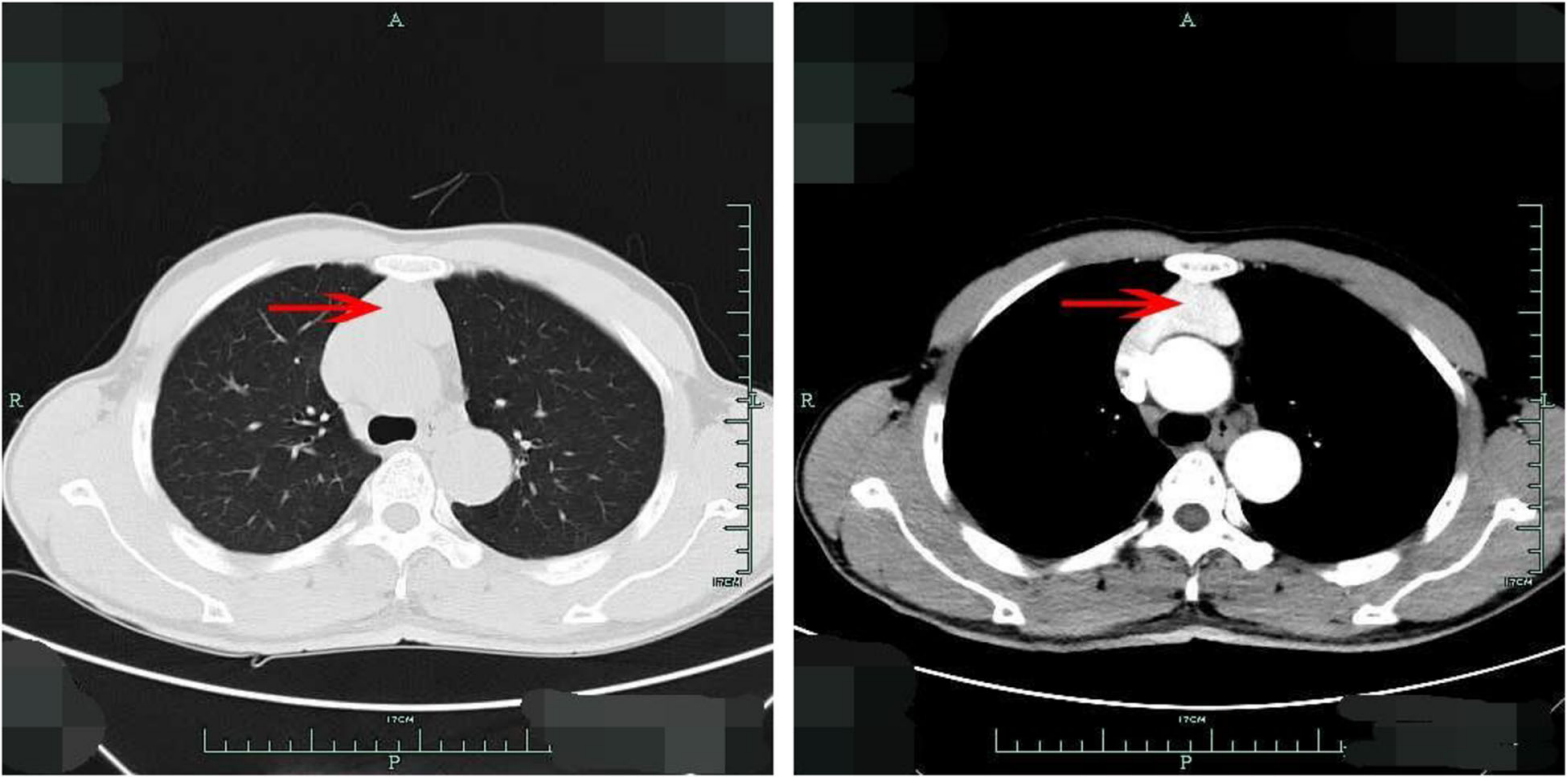
Computer tomography examination of the patient. A large mass of anterior mediastinum was found in CT examination (red arrow), which was enhanced in the artery phase

A 52-year-old man was admitted to our hospital for mediastinal mass found incidentally in health examination. He had no special disease history or any symptoms. No face or upper extremity edema was found in physical examination. Enhanced chest computer tomography (CT) showed a size of 5.6*4.3 cm mass in anterior mediastinum, which was enhanced in the artery phase (Fig. 1), and it had no clear boundary with left innominate vein and SVC. The CT report suggested high possibility of thymoma. Echocardiographic examination did not reveal any abnormality. Thymoma was diagnosed and he received operation in our hospital through median sternotomy on July 2019. During the operation, a dark red and saccular mass was found in anterior mediastinum with slight pulsation. The mass had no clear boundary with left innominate vein and further exploration revealed they were connected with each other. The membrane of the mass seemed to be very thin and easily bled when touched. Left innominate venous aneurysm with part of SVC involved was highly suspected.

**Fig. 2:**
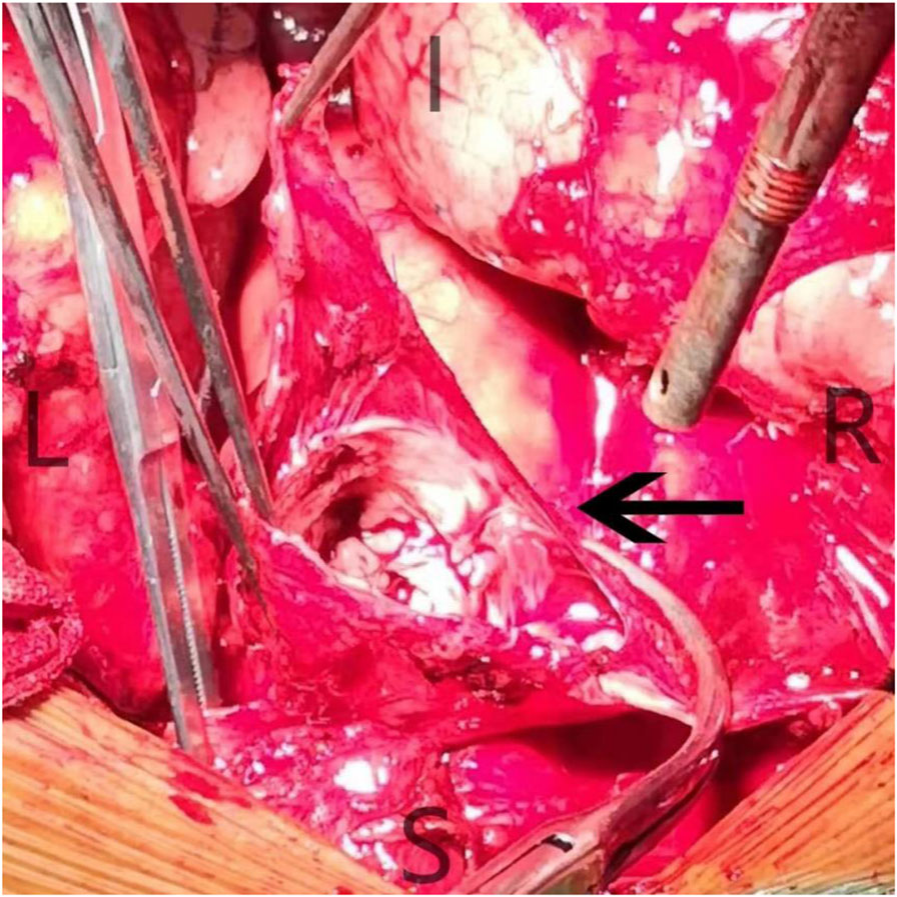
The left innominate venous aneurysm. The left innominate venous aneurysm (black arrow) was resected. ( I: inferior; S: superior; L: left; R: right)

**Fig. 3:**
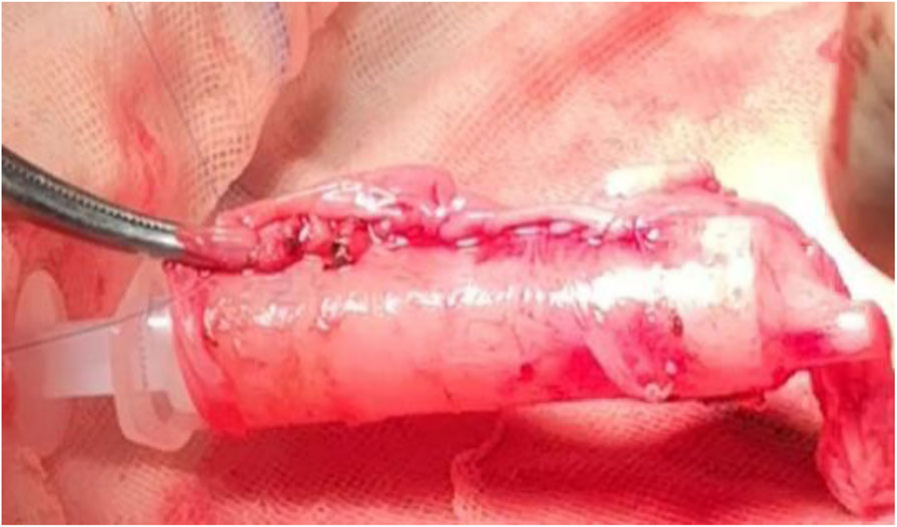
Pericardium made as a tube in shape. Pericardium was wrapped around a needle tube with the smooth surface inside

**Fig. 4:**
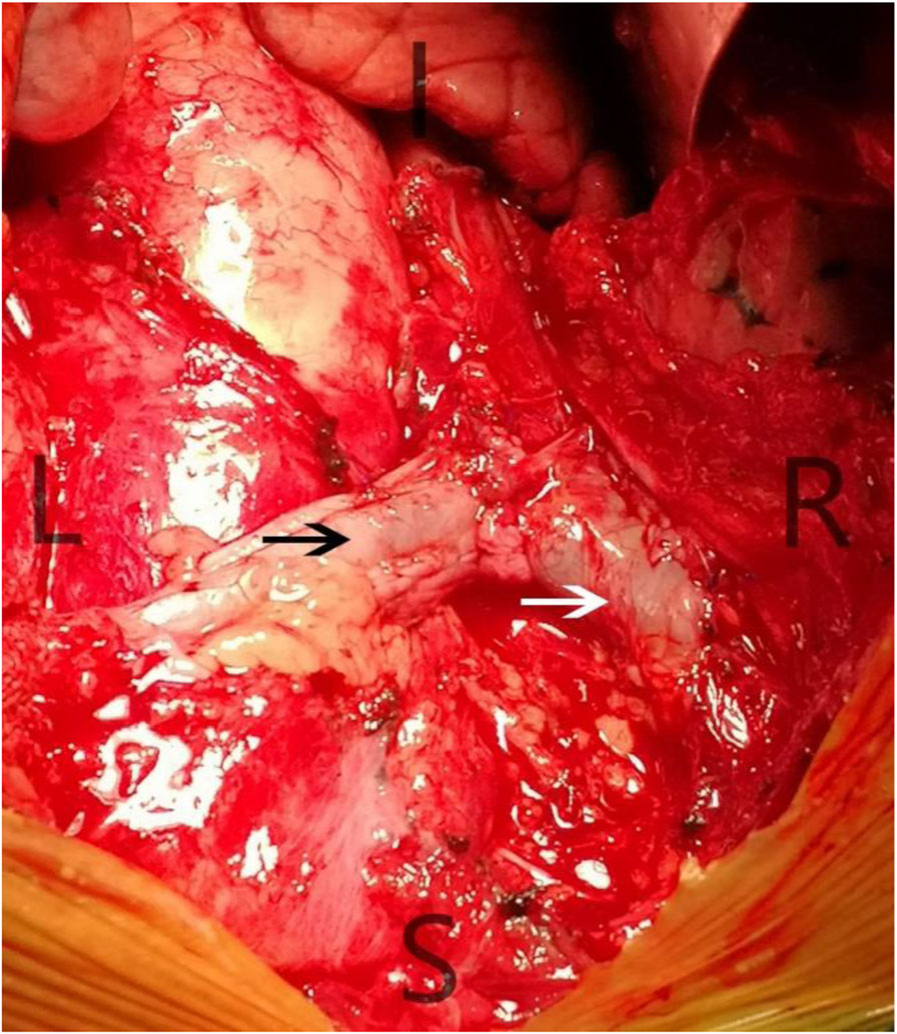
The reconstructed left innominate vein and superior vena cava. The reconstructed left innominate vein (black arrow) and superior vena cava (white arrow) by autologous pericardium. ( I: inferior; S: superior; L: left; R: right)

The venous aneurysm was resected with the proximal and distal end of the left innominate vein blocked (Fig. 2). However, the majority of the left innominate vein wall was involved and only a little part of vessel wall was left after the venous aneurysm was resected, which made the reconstruction of the left innominate vein necessary. Because of the misdiagnosis and lack of abundant preoperative preparation, we decided to take pericardium as material for repairment and reconstruction. We first resected the involved part of SVC with it blocked, and repaired it with a small piece of pericardium. The SVC was unblocked immediately after repairment was finished. Then we took a large piece of pericardium and wrapped it around a needle tube, suturing the two sides to make it a tube in shape with the smooth surface inside (Fig. 3). The tube was first anastomosed to the distal end of the left innominate vein. Then we blocked the SVC again and opened a small hole in the side wall and anastomosed the other side of the tube with SVC. The reconstructed left innominate vein and SVC seemed patent and firm (Fig. 4). Low molecular heparin was used once per day just for routine anticoagulation after surgery. The patient had an uneventful postoperative course and was discharged 6 days after surgery. He did not take anticoagulant drugs after discharge and was satisfied with the treatment therapy. The pathological diagnosis was mediastinal hemangioma. The patient kept in good condition on the last follow up date of December 2019.

## Discussion and conclusions

Left innominate vein aneurysm is very rare and displayed as a mediastinal mass mimicking other tumors such as thymoma [2, 3]. In this case the patient was also misdiagnosed as invasive thymoma before operation which was corrected according to the intraoperative findings. The preoperative CT scan showed no clear boundary between the mass and left innominate vein, and they seemed to connect with each other. We recommend that mediastinal magnetic resonance imaging, computed tomographic angiography and invasive venography can be performed for further evaluation to avoid misdiagnosis once venous aneurysm is suspected or can not be excluded. The correct diagnosis is important because it has impact on the treatment strategy. For patients with small venous aneurysm who have no symptoms, they can be managed with observation only [5]. If follow-up examination shows the venous aneurysm is growing larger and oppresses adjacent structures, or causes symptoms such as cough, chest pain and even dyspnea, or has intraluminal thrombus formation, then surgical intervention is necessary [6–9]. It would be better to choose median sternotomy other than video-assisted surgery due to the risk of rupture of the aneurysm and massive hemorrhage during operation, especially for large vessels. In this case we found the membrane of the aneurysm was very thin and easily bled when touched. If video-assisted thoracic surgery was performed and there would be potential risk for massive bleeding.

Autologous pericardium has already been used in cardiac surgery for reconstruction of heart valve. Quinn et al. reported 62 patients who underwent mitral valve repair with fresh autologous pericardium and obtained excellent long term outcome [10]. B-Khanh Lam reported a patient who suffered from renal carcinoma and received urgent inferior vena cava replacement with autologous pericardium. The patient recovered well after operation without any complication. They believed that autologous pericardium had advantages that it was more malleable, easily available, costless and had better immunocompatibility compared with prosthetic grafts [11]. In this case we used autologous pericardium to repair SVC and reconstruct left innominate vein. The patient had an uneventful postoperative course and kept in good condition after discharge. He did not need to take anticoagulant drugs which was necessary for patients with prosthetic grafts implanted. This was more convenient and safer for the patient after operation.

The SVC was blocked twice in this case we reported. It should be blocked as short as possible because clamping too long may cause brain edema and leads to serious neurological complication. The surgical strategy should be appropriately designed to reduce the blocking time. We repaired the part wall of SVC first and unblock it immediately after the repairment was finished. Then we anastomosed pericardium roll to the distal end of the left innominate vein. The SVC was blocked again when anastomosed with the other end of pericardium roll. The arranged sequence of surgical repairment was to reduce the continuous blocking time of SVC. However, the maximum safe clamping time for SVC remains unclear. If SVC clamping time was estimated to be long in inexperienced centers, venovenous shunt technique can be considered. Dai et al. reported two kinds of venovenous shunt, external and internal. They connected internal jugular and femoral vein before surgery with cannula which was called external venovenous shunt. The internal venovenous shunt was performed during surgery between right brachiocephalic vein and right atrium. Venovenous shunt can reduce the central venous pressure and median SVC clamping time reached 75 min without any complications as reported [12].

In summary, for patients with mediastinal mass, magnetic resonance imaging, computed tomographic angiography and invasive venography can be performed for further evaluation once venous aneurysm is suspected or can not be excluded. Median sternotomy is recommended for aneurysms of large vessels because of the potential risk of massive bleeding and the possible challenges faced during operation such as need for reconstruction of large vessels. Using autologous pericardium to repair and reconstruct large veins is a good choice which is safe and costless.

## Data Availability

The clinical data can be achieved in the electronic medical record system in our hospital.

## Abbreviations

SVC: Superior vena cava
CT: Computer tomography
